# Purification of Sulphate Leach Liquor of Spent Raneynickel Catalyst Containing Al and Ni by Solvent Extraction with Organophosphorus-Based Extractants

**DOI:** 10.1100/2012/286494

**Published:** 2012-02-14

**Authors:** Satunuri Venkateswar Rao, Dong Hyo Yang, Jeong Soo Sohn, Soo-Kyung Kim

**Affiliations:** Urban Mining Research Team, Korea Institute of Geosciences and Minerals Resources (KIGAM), 92 Gwahang-no, Yuseong-gu, Daejeon 305-350, Republic of Korea

## Abstract

Solvent extraction (SX) separation of Al from Ni sulphate leach liquor (LL) of spent Raneynickel catalyst containing 0.12 M Al and 1.448 M Ni using organophosphorus extractants has been investigated. Optimization of process conditions includes aqueous pH, extractant concentration, phase ratio, and stripping. Comparison of Al extraction efficiency with 0.45 M extractant concentration for TOPS 99, PC 88 A, and Cyanex 272 at an equilibrium pH of 2.23 was 81.8%, 98.6%, and 75%, respectively. The corresponding coextraction of Ni was 0.65, 0.6, and 0.9. Among the three extractants screened, PC 88A showed better extraction efficiency for Al at lower pH values than the others. Using 0.45 M PC 88 A, extraction isotherm was obtained at an aqueous-to-organic (A : O) phase ratio of 1 : 1–3 and O : A ratio of 1 : 1–5, which predicted possible separation of Al in 2 stages at A/O ratio of 2. Quantitative stripping was achieved by H_2_SO_4_.

## 1. Introduction

Nickel is primarily used as an alloying metal. The other uses of Ni are in electroplating batteries and as catalysts. Sulfidic, oxidic nickel ores and various nickel bearing secondary materials, such as super alloy scrap, spent batteries, and catalysts are potential sources for Ni production. Preparation of Ni-based catalysts generally involves either an alloy of Ni-Al, impregnation of Ni over the oxide supports of Al, Si, Mg, in the matrix of Al-Mg as hydrotalcites. Ni-based catalysts are widely used in petroleum, petrochemical, and pharmaceutical industry. After the usage of catalyst for a certain period of time, its activity reduces, at this stage, it is considered as spent. Spent catalysts are harmful to the environment due to the presence of soluble/leachable organic and inorganic compounds [1, 2].

 A number of hydro- and pyrometallurgical processes have been proposed for metals recovery from spent catalysts, fly ash, and boiler ash. In general, untreated or pretreated spent catalyst materials are leached with mineral acids alone or their mixtures, sometimes in the presence of additives such as hydrogen peroxide, for the dissolution/leaching of valuable metals. Invascanu and Roman [3], Al-Mansi and Abdel Monem [4], and Abdel-Aal and Rashad [5] optimized the conditions for extraction of Ni using H_2_SO_4_ as leaching reagent. Other authors like Loboiko et al. [6], Vicol et al. [7], and Chaudhary et al. [8] studied leaching for recovery of Ni by using different reagents. Leach liquors obtained by acid treatment of these spent catalysts results in leach solutions containing major content of Ni and less quantities of Al, Mg, and Si. The metals are recovered as mixed solutions and then nickel separated is through conventional separation techniques such as precipitation, solvent extraction, and ion exchange, leaving other metals. In the present study, leach liquor obtained through dilute sulphuric acid leaching of spent Raneynickel catalyst containing about 85 g/L Ni and 3.25 g/L Al with a pH of 0.7 was used Lee et al. [9] for the purification of NiSO_4_.

The separation and recovery of Al by solvent extraction has been studied by several authors under different experimental conditions and in the presence of different associated metals. Acidic organophosphorus extractants are recommended by Meyer Fekete. [10] for the Al extraction and separation of Fe and Al from acidic solutions similar to the pickling bath solution. Mishra and Dhadke, [11], examined Cyanex 921 diluted in cyclohexane for the separation of Be(II) and Al(III). Beryllium extraction was effective in the pH range 8–10 and Al extraction in the pH range 4.5–5.5. SX of Al(III) from aqueous succinate media using n-octylaniline in toluene resulted in maximum Al extraction in the pH range 5.9–6.2, Shilimkar et al. [12]. SX of trivalent Al and Ga from alkali solution containing tartaric acid by trioctylmethylammonium chloride, Hiroshi and Yasushi [13], from weakly acidic medium with di-*n*-butyldithiophosphoric acid (DBTPA) and di-(2-ethylhexyl) dithiophosphoric acid (DETPA) in kerosene, in the presence and absence of alcohols and tri-*n-*butyl phosphate (TBP) Tóth et al. [14] from Co, Ni, and Mg, Tsakiridis and Agatzini-Leonardou [15] have been reported. Two-stage leaching of activated spent HDS catalyst followed solvent extraction of Al using Cyanex 272 as an extractant for the separation of Al from Ni and Co, Park et al. [16]. Solvent extraction and separation of Al has been reported by many researchers using various extractants Zaki et al. [17]; Nagib et al. [18].

 The present research work is aimed at the solvent extraction of Al by organophosphorus-based extractants, in the presence of Ni from sulphate leach liquor of spent Raneynickel catalyst. Experimental analysis of the data was used to determine the main effects and interactions of the chosen factors and select the optimum conditions. The factors studied were effect of equilibrium pH, screening of extractants, A : O phase ratio, and counter-current extraction simulation for Al. Studies on stripping of metal loaded organic phase and regeneration and reuse of extractant were also reported.

## 2. Experimental

### 2.1. Apparatus and Reagents

A Perkin-Elmer Model A 300 atomic absorption spectrophotometer (AAS) and a digital Digisun pH meter (model DI 707) were used. TOPS 99, Talcher Organo Phosphorus Solvent an equivalent of di (2-ethylhexyl) phosphoric acid from Heavy Water Plant, Talcher, India, PC 88 A (2-ethylhexyl phosphonic acid mono 2-ethylhexyl ester, Daihachi, Japan) and Cyanex 272 Bis (2,4,4-trimethyl pentyl phosphinic acid), Cytec, Canada, were used as extractants. Distilled kerosene (b.p: 160–200°C) mostly aliphatic (96.2%), was used as diluent. All other chemicals used were analytical grade. Spent Raneynickel catalyst sulphate leached solution containing 0.12 M Al and 1.448 M Ni was used for present study, Lee et al. [9].

### 2.2. Solvent Extraction Procedure

Suitable volumes of aqueous and organic phases (50 mL each) were taken in a 250 mL glass reactor, which had a provision to insert the electrode and measure the pH of the aqueous phase during extraction experiments. Both the phases were stirred for 5 min (initial experiments showed that equilibrium was reached within 1 min), adjusted to the desired pH values with the addition of 50 wt/vol% NaOH/or H_2_SO_4_ solution. The phases were allowed to separate and the metal concentration in the aqueous phase was measured directly after suitable dilutions by AAS. The loaded organic phases were stripped three times with 2 M H_2_SO_4_ wherever necessary, and the combined strip solutions were analysed for metal values after proper dilution by AAS. All the experiments were carried out at room temperature (25 ± 1°C). The distribution ratio, *D*, was calculated as the ratio concentration of metal present in the organic phase to that in the aqueous phase at equilibrium. From the *D* values, the percentage extraction

(%*E* = *D* × 100/[*D* + (*V*_aq_/*V*_org_)]) where *V*_aq_  and *V*_org_  are the volumes of aqueous and organic phases, respectively, and separation factor (*β* = *D*_Al_/*D*_Ni_) was calculated.

## 3. Results and Discussion

### 3.1. Effect of Equilibrium pH on Extraction of Metals

**Figure 1:**
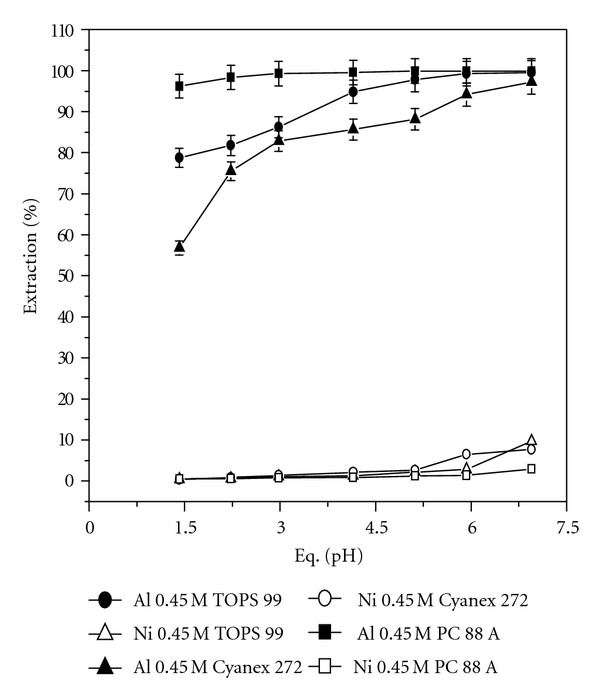
Equilibrium pH on percentage extraction of Al and Ni; conditions: Al = 3.25 g/L; Ni = 85 g/L; reagents = 0.45 M; TOPS 99; PC 88 A; Cyanex 272; equilibrium pH, 1–7.0.

Figure 1 shows the effect of equilibrium pH versus percentage extraction of Al and Ni with 0.45 M concentration of TOPS 99, Cyanex 272, and PC 88 A. In case of TOPS 99 as the extractant in the equilibrium pH range of 1.42 to 7, it was observed that the percentage extraction of Al increased from 78.8 to 94.9% up to an equilibrium pH of 4.15 and thereafter reaches 99.5% at an equilibrium pH of 7. In the equilibrium pH region of 4.15 to 7.0, the coextraction of Ni was increased from 1.3 to 9.8%. Using PC 88 A as the extractant in the equilibrium pH range 1.42 to 5.21, it was observed that Al extraction was increased from 98.2 to 99.9% whereas the co-extraction of Ni increased from 0.5 to 1.3%. Using Cyanex 272 as the extractant in the equilibrium pH range 1 to 7, it was observed that Al extraction increased from 56.8 to 88.5% in the equilibrium pH range from 1 to 4 and finally reached to 97.3% at an equilibrium pH of 7.0 whereas the coextraction of Ni increased from 0.4 to 7.7% in the equilibrium pH region 1 to 7. Among the three reagents, we have selected 0.45 M PC 88 A for further studies on Al separation from Ni at an equilibrium pH of 2.23 (98.6% Al and 0.65% Ni coextraction) as it showed better extraction efficiency of Al than the others.

### 3.2. Effect of Phase Ratio on Al Extraction

**Figure 2:**
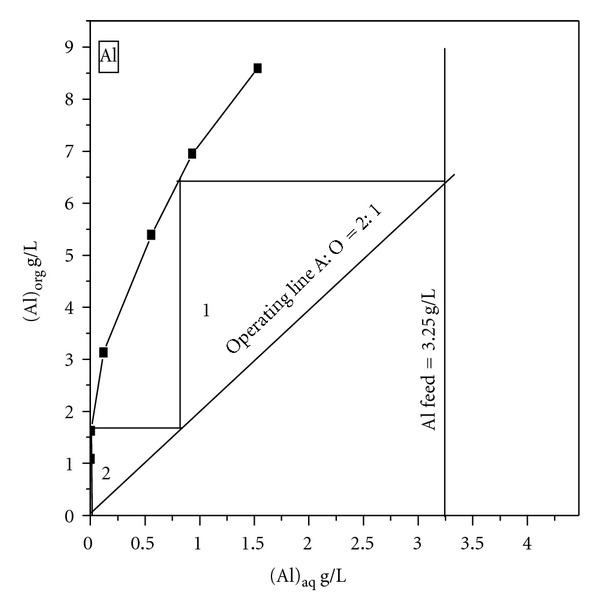
McCabe-Thiele plot for Al extraction. Conditions: Al = 3.25 g/L; Ni = 85 g/L; equilibrium pH, 2.23; A/O, 1/1–3 and O/A, 1/1–5.

To determine the number of stages required at a chosen volume phase ratio for quantitative extraction of Al, the extraction isotherm was obtained at different A : O phase ratios from 1 : 1–3 and O : A phase ratios from 1 : 1–5 by contacting the aqueous feed and organic phase (0.45 M PC 88 A) at an equilibrium pH 2.23 (Figure 2). From the McCabe-Thiele plot, it can be concluded that Al extraction increased from 98.6 to 99.6% with an increase of A : O ratio from 1 : 1 to 3 and decreased from 98.6 to 52.8% at an increase of O : A ratio from 1 : 1 to 5. The results also indicate that Al extraction efficiency of >99.0% can be achieved, if the extraction is performed in two counter-current stages at A : O phase ratio of 2 : 1, where Al extraction efficiency in single stage is about 98.2%.

### 3.3. Counter-Current Extraction Studies for Al Extraction (CCES)

**Figure 3:**
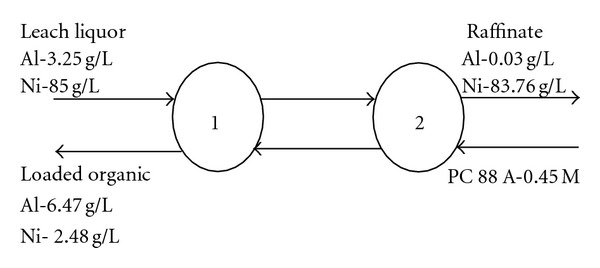
Two stage CCES for Al. Conditions: Al = 3.25 g/L; Ni = 85 g/L; PC 88 A-0.45 M; equilibrium pH, 2.23.

Based on the data obtained from the McCabe-Thiele plot on Al extraction with 0.45 M PC 88 A in kerosene, a two-stage counter current extraction simulation test at A : O 2 : 1 ratio (single stage: 98.2% Al extraction) was carried out at equilibrium pH 2.23 in order to confirm the Al extraction isotherm prediction data. The raffinate and loaded organic outlet streams were collected after the second cycle onwards and analyzed for metal values. The loaded organic (L.O) contains 6.47 g/L Al. The raffinate contained 0.03 mg/L Al corresponding to 99.1% extraction efficiency. Loss of Ni was 1.46% only (Figure 3).

### 3.4. Effect of Stripping Agents

Stripping is the reverse of the extraction, so it should be promoted by these factors that effect extraction negatively, such as acidic, basic, and salt media. Al stripping from loaded organic (L.O) 0.45 M PC 88 A containing 6.47 g/L Al, was investigated using various stripping agents such as HCl, HNO_3,_ and H_2_SO_4_ in the range 0.1–1.0 M at unit phase ratio. The results are presented in Table 1. It was found that stripping efficiency increased with increasing acid concentration and reaches its maximum at 1.0 M. Comparison of stripping data at 1 M acids, it is clear that H_2_SO_4_ is the most effective acid for Al stripping followed by HCl and HNO_3_. Further, the effect of different O : A phase ratios on Al stripping efficiency and number of theoretical stages required for quantitative stripping are presented in Table 2. Al stripping efficiency of 99.6% was achieved in a single stage using 1.0 M H_2_SO_4_. By increasing the O/A ratio to >1, the stripping efficiency decreased and the numbers of stages are required for quantitative stripping increased as expected.

### 3.5. Regeneration and Reuse of the Reagents

**Figure 4:**
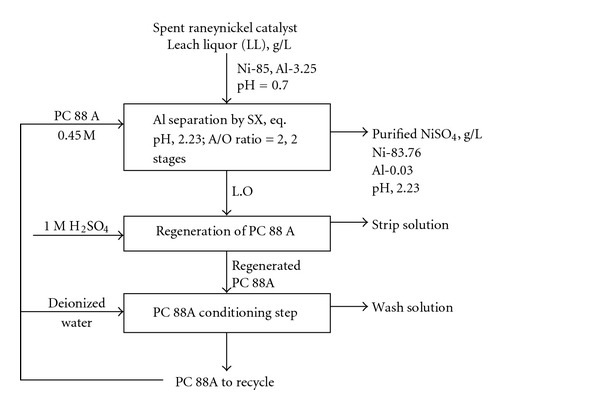
Regeneration and reuse of PC 88 A.

The Al containing organic phases were stripped with acidified distilled water at an equal phase ratio. The stripped organic phases were again washed with distilled water for 2-3 times. The regenerated stripped solvents were reused after washing with deionized water for the determination of extraction efficiency of Al for 5 cycles (Figure 4), and found that extraction and stripping efficiency were the same (standard deviation of ±2%).

## 4. Conclusion

Solvent extraction studies of Al from sulphate solutions by organophosphorus-based extractants establish the dependence of Al extraction on equilibrium pH of the aqueous phase. The results demonstrate that among the phosphoric, phosphonic, and phosphinic reagents, phosphonic acid, that is, PC 88 A is the better reagent for the separation and recovery of Al from Ni from sulphate leach solution of Raneynickel catalyst. Al extraction efficiency of >99% is achieved in two stages at an A : O phase ratio of 2 : 1 and equilibrium pH of 2.23 with PC 88 A. Quantitative Al stripping can be achieved in single stage from loaded organic phase using 1.0 M H_2_SO_4_ by operating stripping at O/A ratio of 1–1.5. Finally, the present methodology can yield high pure Ni LL suitable for production of hydrated NiSO_4_ by evaporation and crystallization.

## Figures and Tables

**Table 1:** Effect of stripping agents on stripping efficiency of Al from loaded organic (L.O; 6.47 g/L).

Stripping agent	Concentration (M)	Al stripping (%)
HCl	0.1	34.6
	0.5	52.3
	1.0	87.4

HNO_3_	0.1	39.0
	0.5	68.9
	1.0	83.5

H_2_SO_4_	0.1	53.9
	0.5	74.3
	1.0	99.6

**Table 2:** Effect of phase ratio on Al stripping from loaded organic (L.O). Al in L.O = 6.47 g/L H_2_SO_4_; 1 M, time: 15 min.

Extractant	Phase ratio (O : A)	Al stripping efficiency (%)	Theoretical number of stages to get >99% efficiency
PC 88 A	1.0 : 1	99.6	1
	1.5 : 1	95.9	2
	2.0 : 1	80.0	3
	2.5 : 1	71.0	4
	3.0 : 1	61.3	5
